# Percutaneous Coronary Intervention With Dual Antiplatelet Therapy in a Patient With Chronic Subdural Hematoma: Novel Approach

**DOI:** 10.7759/cureus.30674

**Published:** 2022-10-25

**Authors:** Aniekeme S Etuk, Celestine I Odigwe, John Andrew Cox, Hercules Panayiotou

**Affiliations:** 1 Internal Medicine, Thomas Hospital Infirmary Health, Fairhope, USA; 2 Internal Medicine, Thomas Hospital Internal Medicine Program, Fairhope, USA; 3 Interventional Neurology, Mobile Infirmary Medical Center, Mobile, USA; 4 Interventional Cardiology, Mobile Infirmary Medical Center, Mobile, USA

**Keywords:** ct (computed tomography) imaging, chronic subdural hematoma (csdh), percutaneous coronary intervention, middle meningeal artery embolization, antiplatelet therapy

## Abstract

Subdural hematoma is a type of brain bleed characterized by the accumulation of blood beneath the dura matter. It usually occurs as a sequela of a traumatic event or following the use of antiplatelets and/or anticoagulants. The clinical presentation may include symptoms like headache, confusion, ataxia, and hemiparesis. However, it may even be asymptomatic, especially in the elderly population. The presence of subdural hematoma is a relative contraindication to antiplatelet therapy because of the associated risk of worsening bleeding. Hence, acute coronary syndrome or conditions requiring antiplatelet therapy presents a management dilemma when they coexist with subdural hematoma.

This paper reports a case of successful use of dual antiplatelets post percutaneous coronary intervention in a patient with spontaneous chronic subdural hematoma. Our patient had a history of coronary artery disease six months prior to stent placement and was on dual antiplatelet therapy. He developed a headache some months later and his neurologist, on evaluating him, made a diagnosis of subdural hematoma, evident on magnetic resonance imaging of the brain. His antiplatelet therapy was discontinued, and he subsequently had a bilateral middle meningeal artery embolization. Following the procedure, a left heart catheterization was done with appropriate interventions for acute coronary syndrome diagnosed at the time of presentation. He was later discharged on dual antiplatelet therapy, followed up on outpatient at scheduled intervals, and was found stable.

This case report suggests that individuals with chronic subdural hematoma who may require antiplatelet therapy can still go on to receive the medication after undergoing a bilateral middle meningeal artery embolization. More observational studies are needed to make this the standard of care.

## Introduction

Subdural hematoma is a collection of blood under the dura mater of the brain [1]. It is more frequently seen in the elderly, with trauma and antiplatelets/anticoagulant therapy being the most common etiology [2]. The incidence in this elderly population is estimated to be about 74 persons per 100,000 [3]. It is believed that the source of bleeding is the rupture of bridging veins into the subdural space [3-5]. The use of antiplatelets and/or anticoagulants remains a relative contraindication in patients with brain bleeds because of associated expansion [2]. We present the first case of percutaneous coronary intervention and dual antiplatelet use in a patient with subdural hematoma after a successful bilateral middle meningeal artery embolization.

## Case presentation

A 68-year-old male with a significant history of coronary artery disease status post two recent drug-eluting stent placements, measuring 3.5 x 38 mm and 3.5 x 16 mm, respectively, six months prior, presented with a complaint of recurrent midsternal chest pain of a day's duration. He described it as a heavy-pressure type of pain, relieved with nitroglycerin. The symptom was associated with shortness of breath. His neurologist discontinued his dual antiplatelet therapy (aspirin 81 mg daily and prasugrel 10 mg daily) a month before the current presentation following a severe headache that on evaluation with a brain magnetic resonance imaging (MRI) scan, revealed subdural hematoma. The patient noted headache to have started four months after stent placement, with a gradual increase in intensity and severity, necessitating a neurology evaluation a month later. Vital signs were blood pressure of 147/99 mmHg and heart rate of 90 beats/min. Physical examinations revealed an elevated body mass index and normal heart sounds with no significant cardiovascular or neurologic examination findings. He has a past medical history of heart failure with a reduced ejection fraction of 30%, end-stage renal disease on peritoneal dialysis, type II diabetes mellitus, gout, hyperlipidemia, and primary hypertension.

**Figure 1:**
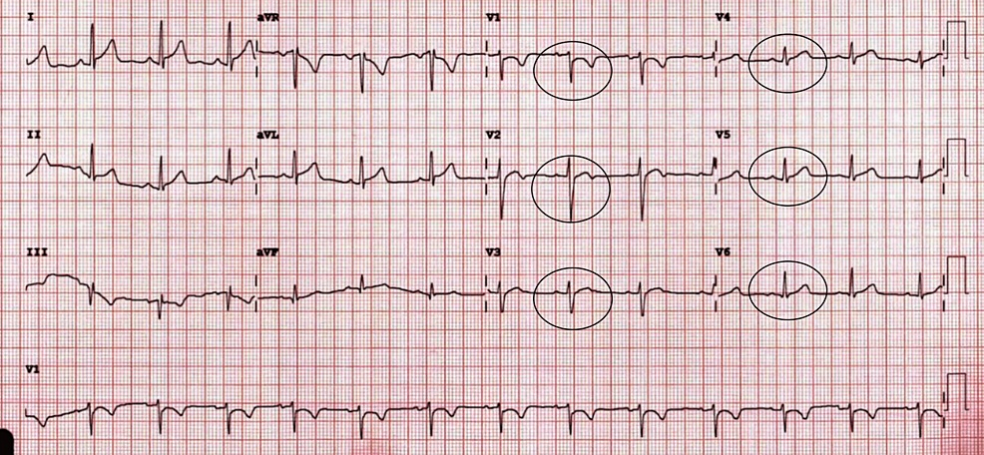
Electrocardiogram showing sinus rhythm with poor R wave progression (see black circle) and non-specific ST changes

**Figure 2:**
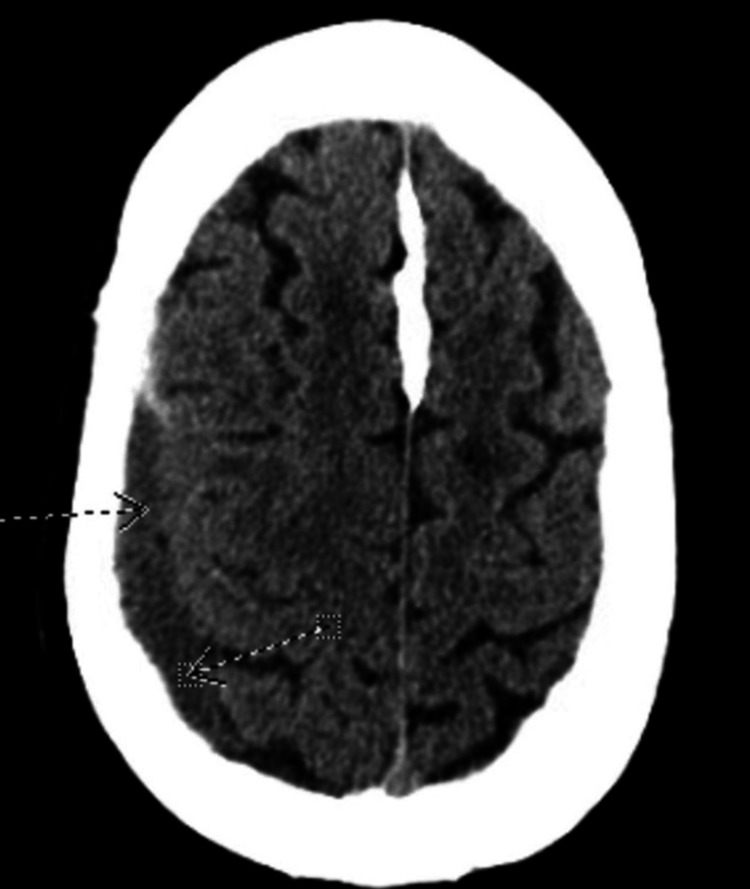
Computed tomography scan of the brain showing a right-sided hypodense collection (see arrows) in keeping with a chronic subdural hematoma

The acute coronary syndrome was suspected based on his symptomatology and history of coronary artery disease with recent stent placement. However, pulmonary embolism and aortic dissection could not be excluded at the time of presentation. The patient had serial electrocardiograms showing a normal sinus rhythm with poor R wave progression and nonspecific ST changes (Figure 1). Serial troponin T was monitored over 24 hours and showed an upward trend, 0.16 ng/ml, 0.19 ng/ml, and 0.25 ng/ml. A computed tomography (CT) scan of the head without contrast showed a small right subdural hematoma appearing to be subacute to chronic with no significant mass effect (Figure 2), similar to earlier MRI scan findings. A repeat MRI was not indicated as the diagnosis of subdural hematoma was already made, and the patient had no worsening neurologic symptoms that would warrant a repeat MRI. A computed tomography angiogram (CTA) of the chest was negative for pulmonary embolism and aortic dissection.

**Figure 3:**
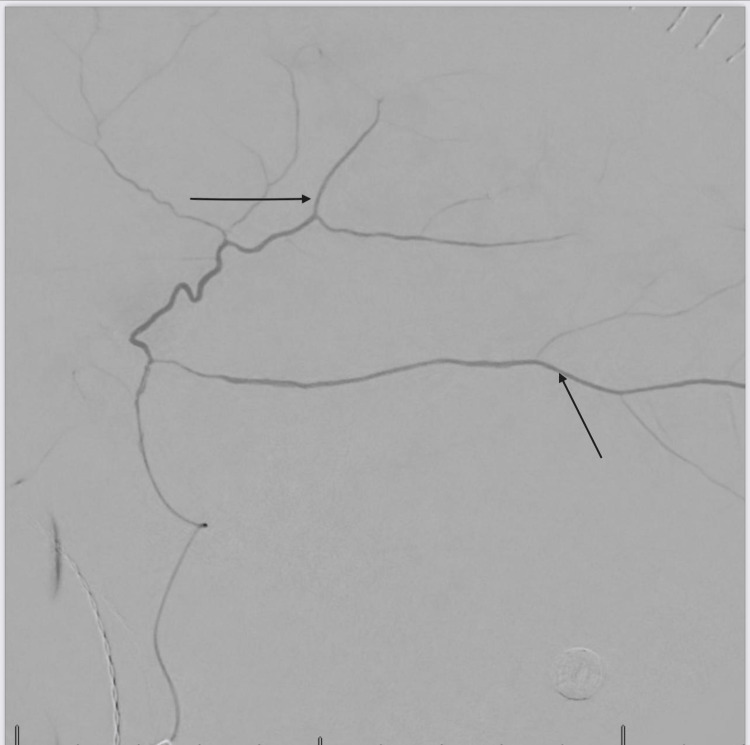
Middle meningeal injection pre-embolization, with distal penetration of contrast (see black arrows)

**Figure 4:**
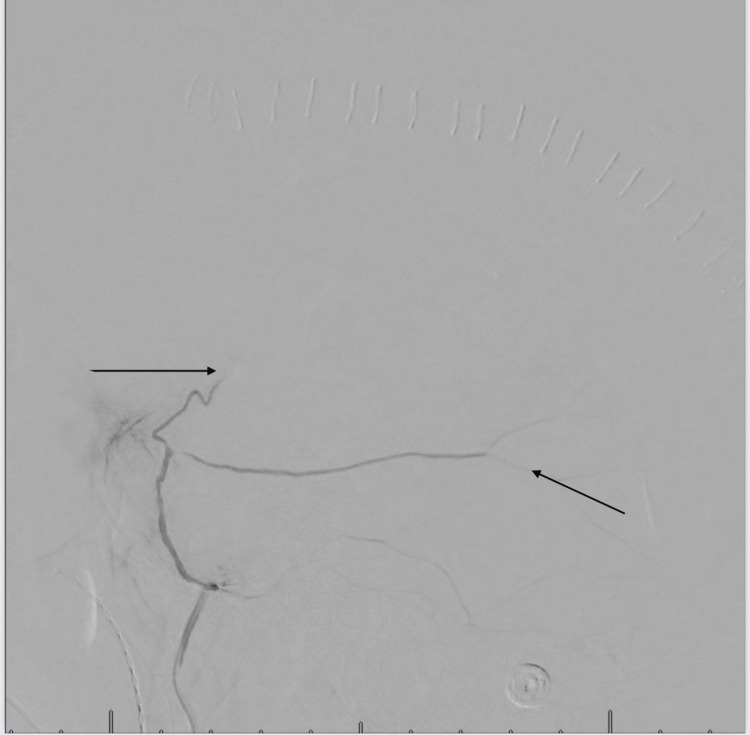
Middle meningeal injection post-embolization, without distal penetration of contrast (see black arrows)

**Figure 5:**
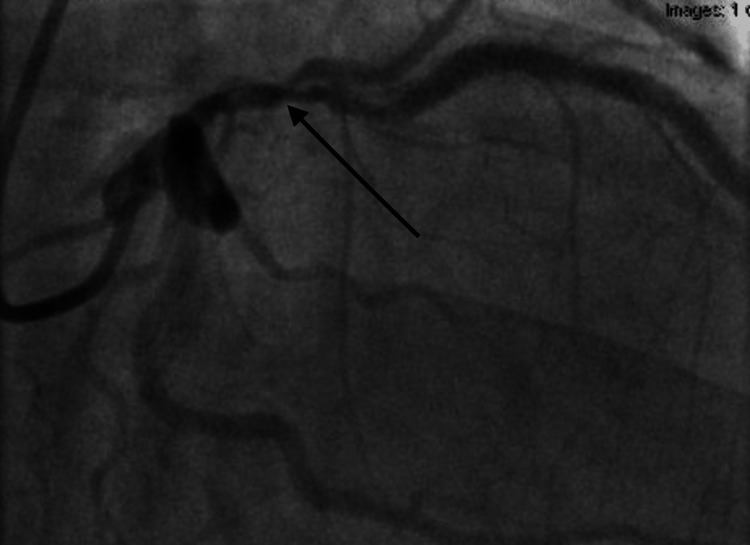
Coronary angiogram showing severe in-stent restenosis to the proximal to mid-left anterior descending artery (see black arrow)

**Figure 6:**
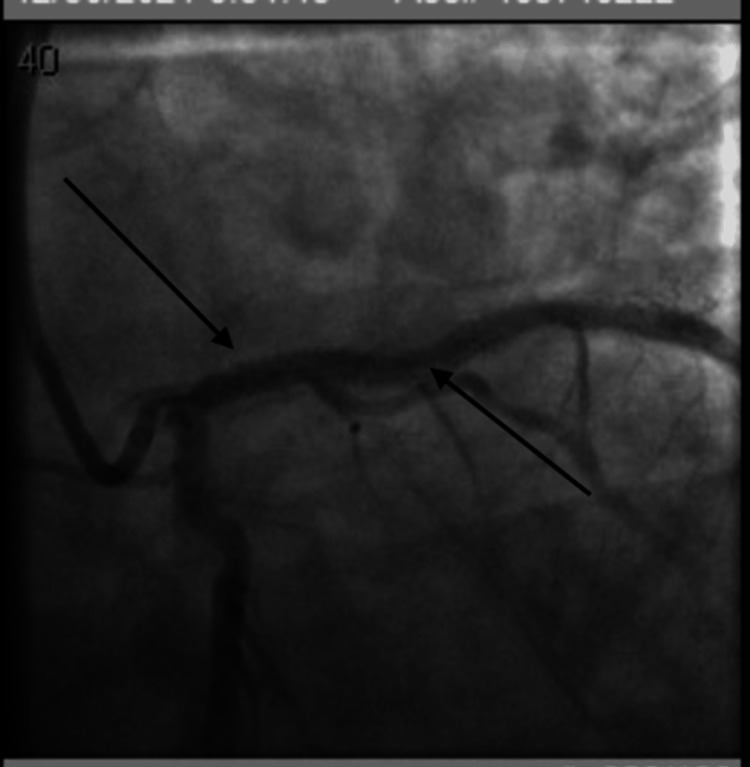
Successful re-stenting to the left anterior descending artery with a 3.5 x 24 mm synergy drug-eluting stent (see black arrows)

**Figure 7:**
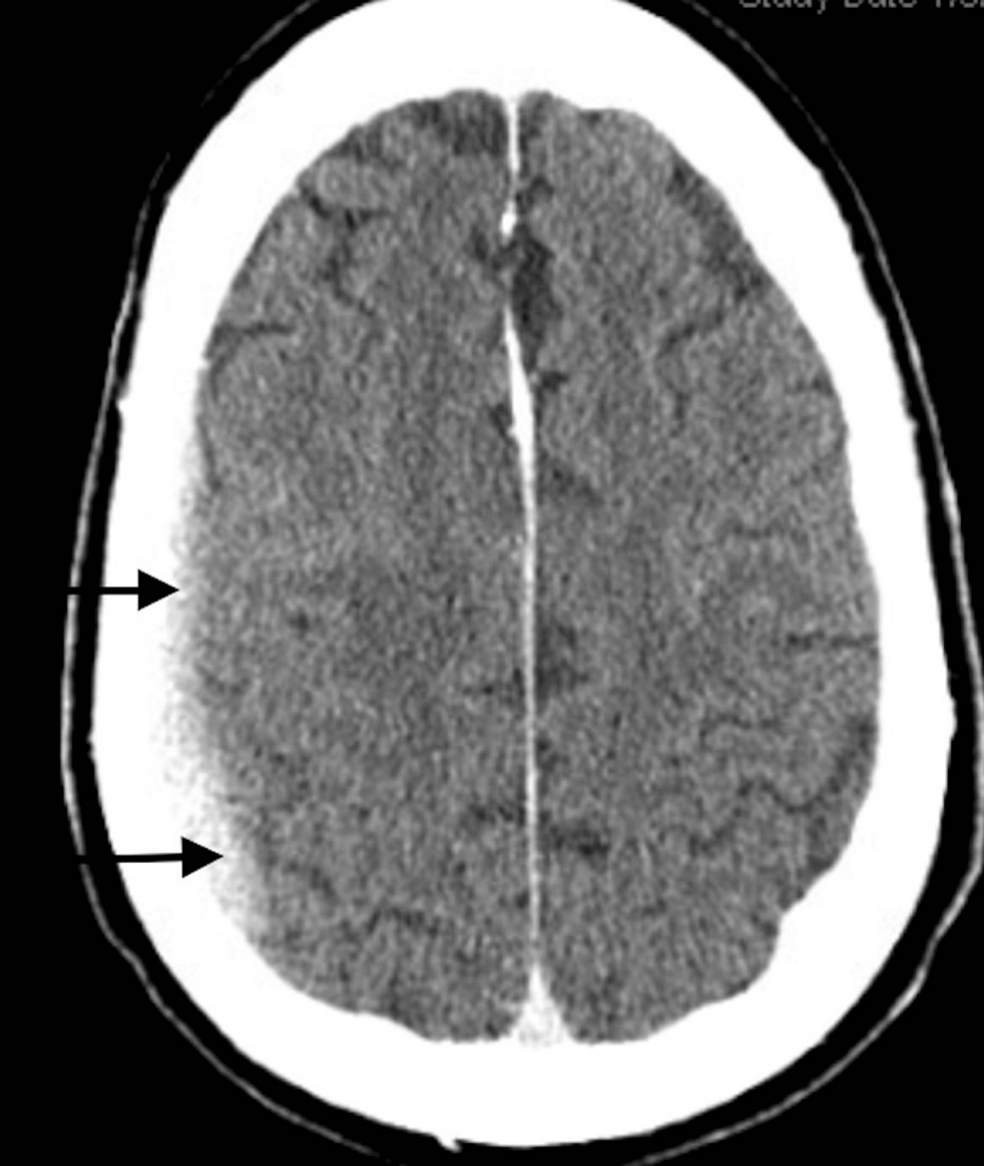
Computed tomography scan of the brain showing a hyperdense collection (see black arrows), which has a contrast effect post-procedure

Management involved a multidisciplinary approach, with expertise from both interventional cardiologists and neurologists. The cardiologist wanted to carry out a left heart catheterization with intervention, however, there were concerns that resuming antiplatelet will worsen the bleeding. Hence, interventional neurology was consulted and carried out a bilateral middle meningeal artery embolization on the patient (Figures 3, 4) using 150 - 250-micron contour polyvinyl alcohol particles. He was managed post-operatively in the neuro-intensive care unit with regular neurological checks, and vital signs monitoring. Three days post-procedure, the patient had a left heart catheterization with findings of severe in-stent restenosis to the proximal mid-left anterior descending artery (Figure 5). This was successfully re-stented with a 3.5 x 24 mm synergy drug-eluting stent, starting at the ostium, extending beyond the first diagonal artery, and overlapping the prior stent (Figure 6). He was commenced on dual antiplatelet therapy with aspirin 81 mg daily and clopidogrel 75 mg daily. The patient spent a week in inpatient service following the coronary artery stenting, with a repeat non-contrast CT scan of the head showing an area of hyperdensity (Figure 7), which is an effect of the contrast used during the middle meningeal artery embolization procedure and is a normal finding following this procedure. He was subsequently discharged home.

**Figure 8:**
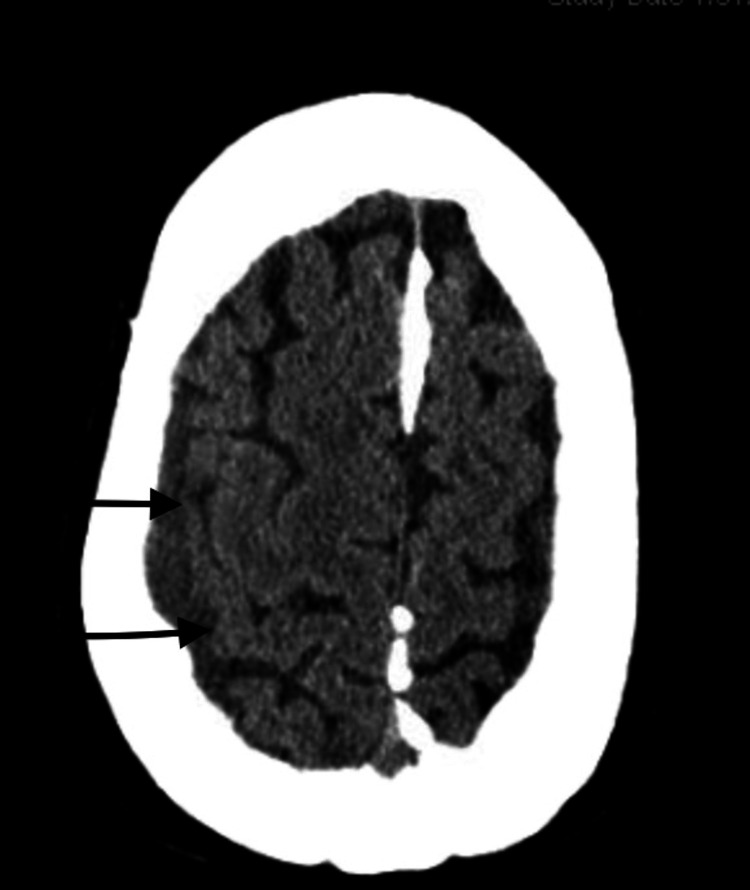
Computed tomography scan of the brain showing a hypodense collection (see black arrows) in keeping with a chronic, right-sided subdural hematoma

**Figure 9:**
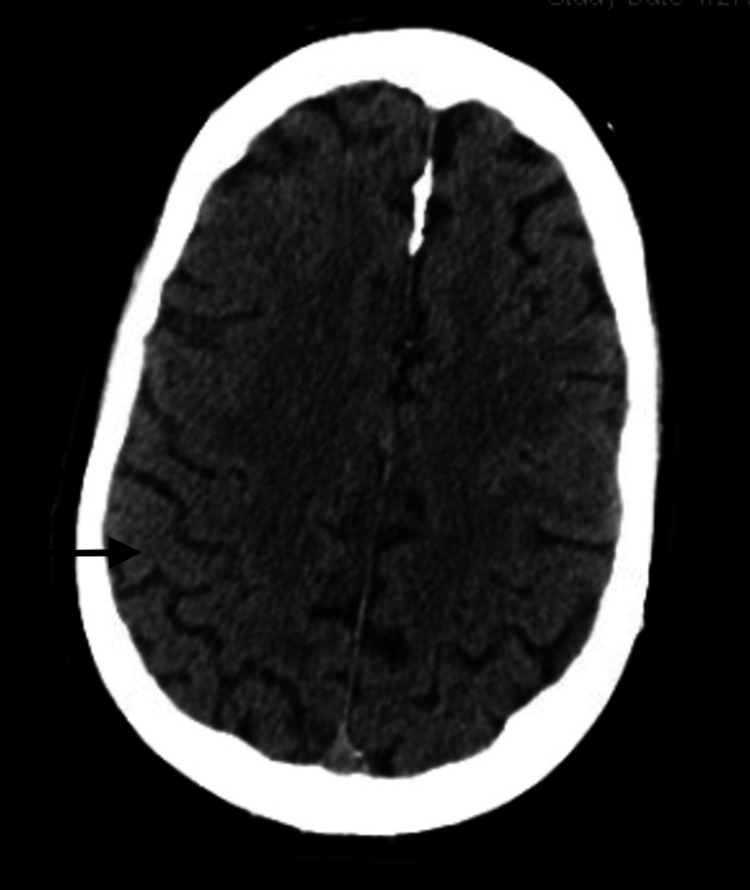
Computed tomography scan of the brain showing complete resolution of subdural hematoma (see black arrow)

A one-month follow-up visit in the outpatient clinic was negative for any neurologic deficit. A non-contrast CT scan of the head was repeated at that visit, with findings of stable right subdural hematoma consistent with previous imaging before discharge (Figure 8). He was also seen six months post-procedure with a CT scan of the brain showing complete resolution of subdural hematoma (Figure 9) despite the continuous use of dual antiplatelet therapy. The patient will be followed up at one-year and two-year time intervals.

## Discussion

A subdural hematoma can be managed either surgically with evacuation or medically with steroids, antifibrinolytics, and statins [3-5]. However, with the advancement in minimally invasive technology, middle meningeal artery embolization has become the mainstay for the stabilization of refractory subdural hematoma [3]. A systematic review and meta-analysis using PRISMA guidelines was carried out to assess the efficacy and safety of middle meningeal artery embolization in comparison with traditional treatments for refractory subdural hematoma [3]. It was found that over 90% of cases treated with middle meningeal artery embolization had good outcomes devoid of complications, 2.8% showed treatment failure, 2.7% required repeat surgical intervention, and 1.2% had embolization-related complications [3].

Antiplatelet therapy has been a relative contraindication in patients with acute or chronic subdural hematoma, as it could worsen bleeding [2]. An Italian case-control study was carried out to ascertain the association between chronic subdural hematoma and antiplatelets/anticoagulants therapy [6]. This was an institutional study with participants aged 60 years and above [6]. There were 345 cases and 1,035 controls, and it was found that those on anticoagulants and/or antiplatelet therapy had higher odds of increased bleeding than those not receiving medication [6]. Our patient presented with symptoms consistent with acute coronary syndrome six months after stent placement and a recent diagnosis of small right spontaneous subdural hematoma about three months post-use of dual antiplatelet therapy. The patient underwent bilateral middle meningeal artery embolization and eventually had a percutaneous coronary intervention with stent placement. He was restarted on dual antiplatelet therapy following coronary artery stent placement. Thus, this will serve as the first reported case of cardiac stenting using antiplatelet therapy in a patient with subdural hematoma.

The etiopathogenesis behind the expansion and recurrence of subdural hematoma stems from the fact that with the formation of a subdural hematoma, cytokine-mediated cascades occur, leading to angiogenesis and neovascularization [3-5]. This capillary network formed results in leakage from the arterial system into the hematoma cavity, thereby worsening blood collection [3-5]. It is worthy of note that there are communications between both sides of the middle meningeal arteries via collaterals in the vertex. Hence, to effectively prevent the expansion of a subdural hematoma, both middle meningeal arteries must be embolized.

Management of similar cases is done based on the preference of the managing physician. Most clinicians prefer conservative management, involving either withholding the use of antiplatelet therapy or continuing the medication while monitoring the patient closely for worsening of bleed [5,6]. These are associated with complications such as in-stent restenosis, subdural hematoma expansion, and neurological deficit, among others [5,6]. Therefore, this is the first reported case of carrying out a middle meningeal artery embolization on a patient with chronic subdural hematoma who is in need of continuous use of antiplatelet therapy and no subsequent identifiable brain bleed.

## Conclusions

Management of subdural hematoma has always posed a clinical dilemma in settings requiring the use of antiplatelet therapy or anticoagulant. The advent of middle meningeal artery embolization has brought a change in the management of chronic subdural hematoma, thus giving room for antiplatelet use when indicated. This case report fills the knowledge gap that though subdural hematoma is considered a relative contraindication to the use of antiplatelet therapy or anticoagulants, middle meningeal artery embolization may help circumvent this in select cases. However, more cases with similar successes and possibly a randomized control trial are needed to further strengthen this claim.
